# Variation in the Involvement of Hippocampal Pyramidal Cell Subtypes in Spatial Learning Tasks

**DOI:** 10.1523/ENEURO.0326-25.2025

**Published:** 2025-11-06

**Authors:** Nadja Sharkov, Tina Sackmann, Nikolas A. Stevens, Janina Kupke, Andreas Draguhn, Ana M. M. Oliveira, Martin Both

**Affiliations:** ^1^Institute of Physiology and Pathophysiology, Medical Faculty, Heidelberg University, Heidelberg 69120, Germany; ^2^Department of Neurobiology, Heidelberg University, Heidelberg 69120, Germany; ^3^Department of Molecular and Cellular Neurobiology, Center for Neurogenomics and Cognitive Research, Vrije Universiteit Amsterdam, Amsterdam 1081 HV, Netherlands; ^4^Interdisciplinary Center for Neurosciences (IZN), Heidelberg University, Heidelberg 69120, Germany; ^5^Department of Molecular and Cellular Cognition Research, Central Institute of Mental Health, Medical Faculty Mannheim, Heidelberg University, Mannheim 68159, Germany

**Keywords:** axon, immediate early genes, learning, morphology; pyramidal neuron

## Abstract

Hippocampal pyramidal cells are involved in spatial coding and memory formation. Recent evidence shows that they can be classified according to the origin of their axon, either emerging from the soma (non-AcD for “nonaxon-carrying dendrite”) or from a proximal basal dendrite (AcD). We have shown that AcD neurons account for ∼50% of CA1 pyramidal neurons and that they integrate excitatory inputs differently. They are less susceptible to perisomatic inhibition and more strongly recruited during memory-related network oscillations with strong inhibitory activity. Here, we tested whether AcD and non-AcD neurons are differentially engaged during distinct stages of spatial learning. We trained mice of either sex on a spatial memory task (m-maze) and quantified c-Fos expression in CA1 pyramidal neurons at different training stages. AcD and non-AcD cells were distinguished by staining the axon initial segment. Across learning stages, dorsal and medioventral hippocampus showed distinct activation patterns. In dorsal CA1, c-Fos expression shifted from a predominant presence in non-AcD cells at early stages to the increased presence in AcD cells at later stages. In medioventral CA1, AcD neurons showed a transient c-Fos expression peak at intermediate stages of the training, accompanied by a progressive reduction of the percentage of AcD cells over time. This reduction was not observable in the dorsal hippocampus. This suggests region- and cell type-dependent recruitment patterns of CA1 pyramidal cells during learning and indicates that the site of axon origin may undergo structural plasticity. In addition, the findings support functional and structural differentiation along the dorsoventral axis of CA1.

## Significance Statement

Neurons with axons emerging from dendrites [axon-carrying dendrite (AcD) cells] represent a morphologically and functionally discernible subpopulation of hippocampal pyramidal cells. Here, we show different involvement of AcD and non-AcD cells during different learning stages and hippocampal subregions. Remarkably, the proportion of AcD cells in the medioventral hippocampus dynamically decreases during learning, suggesting that the site of axon origin is structurally plastic. This discovery challenges the long-standing view of fixed neuronal wiring and identifies the AcD as a site of adaptive structural reconfiguration. Our findings reveal a novel, plastic mechanism for tuning neuronal excitability during learning and highlight the dynamic interplay between morphology and function in hippocampal circuits.

## Introduction

The hippocampus plays a key role in the formation of spatial, episodic and declarative memory. These processes have been extensively studied in behavioral experiments with rodents solving spatial tasks (O'Keefe and Conway, 1978; Wisden et al., 1990; Leutgeb et al., 2005). During exploration of an environment, they form place cells, i.e., pyramidal cells that exhibit action potentials in distinct places, forming representations of specific locations (O'Keefe and Dostrovsky, 1971). The formation of such neurons with transiently stable spatial firing behavior depends on the integration of multiple excitatory synaptic inputs to the dendrites and activity-dependent plastic changes in the participating synapses. In a classical view, the pyramidal cell has multiple dendrites that are connected to the soma and an axon that is also directly connected to the soma. Thus, dendritically generated postsynaptic potentials have to travel through the soma to reach the axon initial segment (AIS) where they are integrated and potentially trigger an action potential (London and Häusser, 2005). Active dendritic mechanisms and inhibitory synapses can additionally modulate the dendritic potentials on their way to the AIS. Above all, perisomatic inhibition can effectively and uniformly modulate the signal flow to the axon. This is of particular importance as perisomatic inhibition is strongly activated during hippocampal network oscillations that occur during memory formation and consolidation (Ellender et al., 2010; Gulyás and Freund, 2015).

We have recently shown that a considerable fraction of hippocampal pyramidal cells (20–60%, depending on region) have an axon that originates from a basal dendrite (Thome et al., 2014). With this morphology, the axon-carrying dendrite (AcD) largely evades perisomatic inhibition and thus forms a privileged input channel for action potential generation (Thome et al., 2014). The functional impact of this deviation from canonical morphologies is particularly apparent during network oscillations with strong perisomatic inhibition, such as sharp-wave–ripple (SPW-R) oscillations (English et al., 2014; Geschwill et al., 2020). Consequently, AcD cells have a higher firing probability than non-AcD cells in vivo during SPW-R, while there is no difference in network states with less prominent perisomatic inhibition (Hodapp et al., 2022). Pharmacological and computational approaches showed that the preferred firing of AcD cells is indeed due to the combination of the remote location of the axon origin and perisomatic inhibition (Hodapp et al., 2022). Importantly, SPW-R are involved in the consolidation and retrieval of memories, and neurons that experience learning-related plasticity are more prone to be involved in ripple oscillations than other cells (Girardeau et al., 2009; Ego-Stengel and Wilson, 2010; Jadhav et al., 2012; Mizunuma et al., 2014). Thus, the peculiar features of AcD cells may be reflected in unique network integration and memory-related activity of this group of pyramidal cells. In line with this hypothesis, we recently found that AcD cells in CA1 receive stronger inputs from the contralateral CA3 region than non-AcD cells (Stevens et al., 2024). As shown by others, these connections are involved in spatial reference memory and novelty detection (Adaikkan et al., 2022; Jordan et al., 2022). Together, this implies that AcD cells might play a specific role in learning processes.

To explore the different participation of AcD and non-AcD cells in learning and memory formation, we trained mice in a spatial learning paradigm that requires both spatial working memory and long-term spatial reference memory formation (Karlsson and Frank, 2008; Jadhav et al., 2012). Female and male mice learned to perform a spatial memory task on an m-maze over several days. Neurons involved in such learning processes are expected to express immediate early genes (e.g., *c-fos*) shortly after the exposure to a learning paradigm (Kaczmarek, 1992; Nikolaev et al., 1992; Dragunow, 1996). We therefore analyzed the expression of c-Fos at different stages of the learning task (3, 5, and 7 d, respectively) and sorted c-Fos-expressing cells in AcD and non-AcD neurons. Our findings show that c-Fos expression follows peculiar time courses in different portions of the hippocampus and differs between AcD and non-AcD cells. We also report that the proportion of AcD cells decreases during the time course of the experiment, indicating that the location of the axon in CA1 pyramidal cells can change during a prolonged learning process.

## Materials and Methods

### Animals

Experiments were performed on Thy1-GFP-M mice (JAX stock #007788, Jackson Laboratory) of either sex (29 male, 25 female mice). Thy1-GFP-M mice express GFP in sparse subsets of neurons in the hippocampus yielding a bright Golgi-like stain of individual cells and allowing for subcellular morphological analysis of these cells. All activities were carried out according to the Federation of European Laboratory Animal Science Association guidelines. Animal procedures have been approved by the federal government of Baden- Württemberg (G-62/19 and G-141/21). Animals were kept in Scantainers (Scanbur BK A/S, Denmark) with a 12 h light/dark cycle. During the initial habituation to the experimenter and experimental room, the mice received food and water *ad libitum*. To motivate the animals to carry out the behavioral experiments, they were slightly food deprived to reach 85–90% of the initial body weight. The age of the animals ranged from Postnatal Day (P)60 to P80 by the beginning of the m-maze paradigm.

### Habituation

All mice were habituated to the room for behavioral experiments and to the experimenter prior to the beginning of the spatial memory training. The animals were brought into the behavioral room for half an hour and habituated to the experimenter for ∼15 min. Habituation took ∼1–2 weeks, after which they exhibited a notable decrease in restraint when traversing on the experimenter's hand. During the last 3–4 d of the accommodation phase, the animals were transferred to a separate home cage to begin with the food deprivation.

### M-maze paradigm

**Figure 1.**
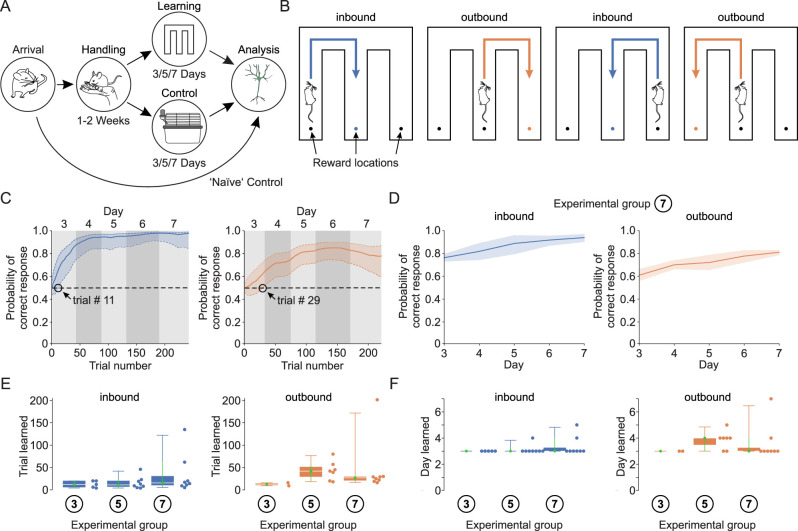
Experimental design and learning curves. ***A***, Mice were habituated by handling over 1–2 weeks. The experimental group performed 2 d of forced trials with subsequent 1, 3, and 5 d of free trials. Control animals were present in the experimental room in their home cages but did not perform any behavioral tests. After 3, 5, or 7 d of experiment, animals were killed, their brains were removed, and the c-Fos expression and morphology were analyzed (Fig. 2). ***B***, Schematics of m-maze rules for successful trials. ***C***, Exemplary learning curves of one animal for inbound (left) and outbound (right) trials over several days. The mouse learned the inbound part on Day 3 on the 11th trial and the outbound part on Day 3 on the 29th trial. ***D***, Medians and 95% confidence intervals of the Day 7 experimental group. ***E***, Quantification of the trial by which the animals learned the inbound (left) and outbound (right) part of the experiment. ***F***, Quantification of the day by which the animals learned the inbound (left) and outbound (right) part of the experiment. ***E***, ***F***, Note that some animals from the 3 and 5 d experimental group did not learn the task in time. Only those animals are plotted and quantified that succeeded in learning the inbound or the outbound task, respectively.

The m-maze (Fig. 1*A*,*B*) measured 52 × 52 cm (Embray and Klumpp, 2024; Klumpp et al., 2025). Individual arms were 10 cm wide with a 1 × 1 cm rim. The maze was raised on a stand to a height of 1 m. Light sensors were fixed near the choice point (decision between two potential paths) and the reward points (at the end of each branch). Signals from these sensors were used for automatic opening/closure of gates during the training phase and for reward delivery (Fig. 1). Food dispensers provided a drop of condensed milk whenever a mouse solved the task successfully. At the choice point, two motorized gates were attached which were used on the first 2 d of the behavioral experiment (forced trials). During the forced trials, the animals received a food reward at the end of each arm. They are intended to habituate the animals to the maze and ensure that they learn that they receive food rewards at the end of the arms. Different distal visual clues were pinned on the walls around the m-maze. Ambient light was set to 20 lux. The animals were food deprived in order to maintain 90% of their individual initial body weight and thus motivate them to participate in the m-maze experiment. At the beginning of the experiment, a mouse was placed at the end of the left arm. Animals could freely explore the maze for 15 min each day. Typically, the number of trials per day increased while the animals learned the paradigm. Animals had to perform the following task (Fig. 1*B*): move from the left to the middle arm (inbound trial), then move from the middle to right arm (outbound trial), move back from the right arm to the middle arm (inbound trial), move from the middle arm to the left arm (outbound trial), and so forth. Thus, each new trial started at an “old” reward point from where the animal had to move to the correct new reward point. If the animal performed the trial correctly, it received 15 µl of condensed milk mixed 1:1 with water for a final concentration of 4% fat, 10% fat-free dry milk, and 27% sugar. If the animal performed the trial incorrectly, no reward was given. In both cases, whether the attempt was correct or incorrect, the new trial began at the animal's current position without interruption or forced waiting time.

Inbound trials are mostly related to spatial reference memory, while outbound trials also involve working memory. After 2 d of forced trials and habituation to the maze, the mice had to solve the task on their own, with all gates staying open (free trials). To examine different stages of learning, different groups of mice were analyzed after 3, 5, and 7 d on the m-maze (including the 2 d of forced trials). Additionally, control animals were handled for the same amount of time and were present in the behavior room but did not perform the m-maze task. As an additional control, we used naive mice that were neither handled nor performed the tasks (Fig. 1).

Mice were video-monitored during the whole behavioral experiment to monitor exact position and movement, to evaluate learning progress, and to control the motorized gates and feeders. Data were acquired with the software “Syntalos” (Klumpp et al., 2025).

### Immunohistochemistry

Mice were perfused under deep anesthesia (ketamine/xylazine, 120 and 16 mg/kg) with polychlorinated biphenyl (PBS) and subsequently with 4% paraformaldehyde (PFA). The perfused brain was removed from the skull and stored at 4°C in a 2% PFA solution overnight. After fixation, it was stored in PBS at 4°C.

The brain was cut into 100-µm-thick slices using a vibratome (Leica VT1200, Leica Biosystems). The left hemisphere was sliced horizontally for analysis of the medioventral hippocampus, while the right hemisphere was sliced coronally for analysis of the dorsal hippocampus.

Slices were permeabilized with 0.1% Triton X-100 in PBS for 5 min. Then, the slices were washed with PBS and blocked with a solution consisting of 8% normal goat serum (NGS) and 0.3% Triton X-100 in PBS for 50 min. After another washing step in PBS (three times, 5 min each), the slices were incubated with the primary antibody overnight (PBS with 2% NGS and 0.3% Triton X-100; for specification and concentration of antibodies, see below). The next day, the slices were washed in PBS (three times, 5 min each) and incubated with the secondary antibody (PBS with 2% NGS and 0.3% Triton X-100; for antibody concentration, see below) for 2 h.

The slices were then transferred onto slides and sealed with DAPI solution and a cover glass.

### Antibodies

The following primary antibodies were used: rabbit anti-c-Fos (1:1,000; #226 003; Synaptic System) and guinea pig anti-AnkG (1:1,000; #386 005; Synaptic System).

The following secondary antibodies were used: anti-rabbit Cy3 (1:500; Cy3 anti-rb; 111-165-003; Dianova) and anti-guinea pig Alexa Fluor 647 (1:1,000; Alexa Fluor 647 anti-gp; A21450; Invitrogen by Thermo Fisher Scientific).

### Confocal laser scanning microscopy

Imaging was carried out on a C1 and A1R Nikon confocal microscope (Nikon Imaging Center Heidelberg). For detailed analysis, a 60× oil immersion objective (1.4 NA) was used. Images were analyzed in ImageJ (Wayne Rasband, NIH open source).

### Data analysis

**Figure 2.**
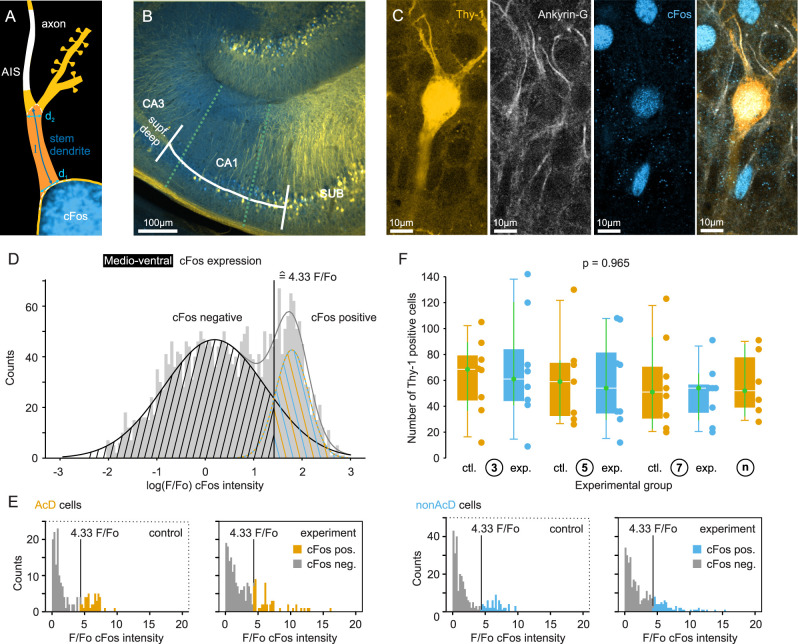
Quantification of the c-Fos expression and classification into AcD and non-AcD cells. ***A***, Scheme of the parameters deﬁning the class of AcD cells. AcD cells have a stem dendrite longer than 2 µm and a mean diameter (*d*_1_ + *d*_2_)/2 smaller than its length l. ***B***, Scheme of the range of analysis in the medioventral hippocampus. ***C***, From left to right, Example images of intrinsic GFP expression in Thy-1 mice, counterstained Ankyrin-G to identify the AIS, counterstained c-Fos, and an overlay. ***D***, Histogram of the normalized c-Fos fluorescence values of all cells from all groups in the medioventral hippocampus. The threshold for c-Fos-positive cells was defined as the intersection of the two log-normal distributions, corresponding to a *F*/*F*_0_ of 4.33. ***E***, Example histograms for AcD cells and non-AcD cells of experimental and control Day 3 in the medioventral hippocampus. The gray values are classified as c-Fos-negative cells; the colored values (exceeding *F*/*F*_0_ of 4.33) are classified as c-Fos positive. ***F***, The number of Thy-1-/GFP-positive cells in the same investigated volume of medioventral hippocampus within the different experimental groups. There was no systematic difference, indicating that Thy-1/GFP expression is not correlated to the behavioral paradigm.

Morphological analysis and classification into AcD and non-AcD cells was performed on three-dimensional image stacks using ImageJ (Wayne Rasband, NIH, open source) and the “Simple Neurite Tracer” plugin (Arshadi et al., 2021). For the medioventral hippocampus, we analyzed a volume of 210 × 210 × 30 µm in five slices. As the number of Thy-1/GFP cells in the dorsal hippocampus was lower, we analyzed 10 slices of the same volume. The origin of the AIS was defined as a strong abrupt increase in ankyrin-G signal. The end of the AIS was defined as the last point of the continuous ankyrin-G signal. Cells were defined as AcD cells if their axon originated from a dendrite and the length of the stem dendrite (i.e., the proximal part of dendrite from soma to branching point of axon) was larger than 2 µm and longer than its mean diameter (Hodapp et al., 2022; Stevens et al., 2024; Fig. 2*A*).

c-Fos intensity was analyzed in ImageJ by choosing an oval ROI with a fixed size of 30.644 pixels fitting to cell bodies of CA1 pyramidal cells under our imaging conditions. Average intensity for each cell was normalized to the background intensity around the pyramidal cell layer of each slice. To do so, 35 random points outside the pyramidal layer were measured and averaged (*F*_0_). c-Fos positive was defined as *F*/*F*_0_ exceeding a threshold value that was defined by the distribution of all cells from all groups by fitting two log-normal distribution (Fig. 2). For medioventral slices the c-Fos threshold was determined to be *F*/*F*_0_ = 4.325 and for dorsal slices to be *F*/*F*_0_ = 2.247.

### Statistics

Data are represented as box plots in which medians are indicated by a line and the box limits denote the interquartile range (25th to 75th percentile). Whiskers of box plots extend to the 2.5th and 97.5th percentile. Confidence intervals for the medians were estimated by means of bootstrapping, i.e., by computing the 2.5th and 97.5th percentile of the distribution of medians from 10,000 resamples. These confidence intervals are plotted as green vertical lines in the box plots. In the text, values are reported as medians and their confidence intervals in the form of median [2.5th confidence value, 97.5th confidence value]. For assessing a potential biological relevance of the percentage of c-Fos-positive cells, we used two different analyses. First, we pooled all AcD or non-AcD cells from different animals of one experimental setting and calculated the odds ratio, effect size, and *p* value based on a contingency table. The rows and columns were number of c-Fos-positive versus c-Fos-negative cells and the control group versus the experimental group. We defined biological relevance if the effect size was >0.2 (which corresponds to at least a small effect) or if the odds ratio was >2 (which means that the probability of an event occurring in one group is at least twice as high as the probability of it occurring in the other group). Fisher's exact test was used to assess statistical significance of contingency tables. As a second analysis, we pooled the cells from individual animals to calculate the percentage of c-Fos-positive cells and tested statistical significance between control and experimental animals with the Wilcoxon rank sum test (corresponds to the Mann–Whitney *U* test). Only if the data were biologically relevant and both the contingency table and the group comparison was statistically significant, we report the data as a meaningful result. Statistical significance for three groups was calculated by the Kruskal–Wallis test, followed by a Dunn–Bonferroni post hoc test if testing reached significance. A *p* < 0.05 was regarded as statistically significant.

## Results

### Time course of learning in the M-maze paradigm

In order to investigate the involvement of AcD and non-AcD cells in learning processes, we used a learning paradigm spanning several days (Fig. 1). The m-maze provides two different tasks depending on the animal's location. Inbound trials (starting from one of the outer arms) involve mainly spatial reference memory, as the animal has to remember the rule to turn into the middle arm and can orient itself by distal cue cards. Outbound trials (starting from the inner arm) require working memory, as the animal has to turn to the arm which was not visited in the preceding trial (Fig. 1*B*). Learning curves were computed using a state-space model of learning (Smith et al., 2004). Learning trial and learning day for each animal were assigned as the first trial and test day at which the 95% confidence interval of the estimated probability of correct performance exceeded and remained above chance level. Inbound trials were typically learnt after 15 trials [9, 62] on the third day [3, 4] (i.e., the first day of free trials), while outbound trials were learnt on Trial 27 [19, 30] also on Day 3 [3, 4] (Fig. 1*C*–*F*). However, learning of the inbound trials further increased until Day 5 and of outbound trials until Day 7 (Fig. 1*D*). Thus, we used Days 3, 5, and 7 to analyze expression of c-Fos in mice that were beginner, advanced, and experienced.

### Classification of cells by c-Fos expression and site of axon origin

Neurons were classified into AcD and non-AcD cells according to Thome et al. (2014; see also Materials and Methods; Fig. 2*A*). Additionally, we stained for the immediate early gene c-Fos as a measure for the activity of individual cells during the preceding behavioral phase (Fig. 2*A*–*C*). Most cells showed some background fluorescence that was apparent when normalizing the c-Fos fluorescence values in the cells to other areas in the same section (Fig. 2*C*). To define a reasonable threshold for c-Fos activation, we quantified the distribution of normalized c-Fos fluorescence intensity values of all analyzed cells (*N* = 2,812 for medioventral hippocampus and *N* = 2,532 for dorsal hippocampus). The respective histograms showed a bimodal log-normal distribution, and we assigned the crossing point of both log-normal curves as threshold between c-Fos-negative and c-Fos-positive cells (Fig. 2*D*). With this approach, the threshold for c-Fos-positive cells in medioventral hippocampus was 4.33 *F*/*F*_0_ and in dorsal hippocampus 2.25 *F*/*F*_0_. Figure 2*E* shows an example of the c-Fos fluorescence intensity distribution of AcD and non-AcD cells of the medioventral hippocampus from one control mouse and one experimental animal on Day 3. The morphological classification was based on the random expression of Thy-1 in CA1 pyramidal neurons. To rule out the possibility that the learning paradigm had an influence on the expression of Thy-1, we compared the number of Thy-1–positive cells found in the same volume in each group (Fig. 2*F*; see Materials and Methods for the volume analyzed). No difference was observed in either the medioventral or dorsal areas (*p* = 0.965 for the medioventral hippocampus and *p* = 0.935 for the dorsal hippocampus).

### Comparison of the number of c-Fos-positive AcD and non-AcD cells in experimental versus control animals

**Figure 3.**
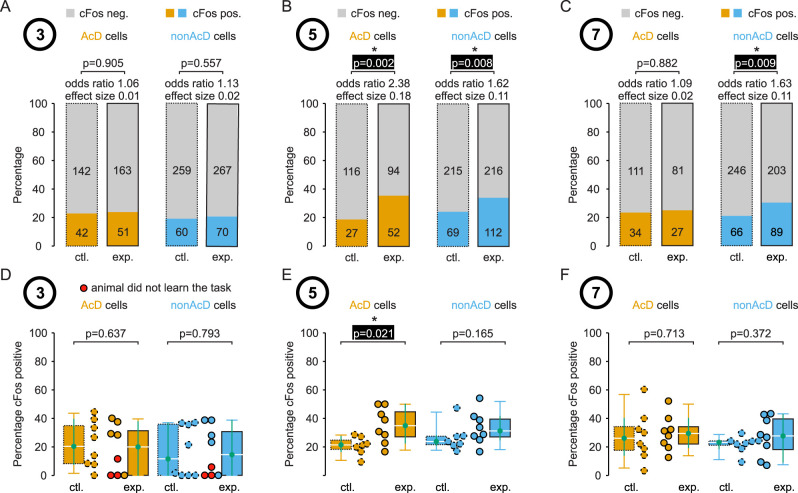
Quantification of cells expressing c-Fos in the medioventral hippocampus. Cells from three different experimental groups are analyzed: animals performed the behavior task for 3 d (***A***), for 5 d (***B***), or for 7 d (***C***). All cells from eight animals from each group were pooled, and the percentage of c-Fos-positive cells was quantified. Respective control groups were handled and present in the experimental room but did not perform the behavioral task. The percentage of c-Fos-positive AcD cells from the 5 d experimental group was ∼2 times larger than in the control group (Fisher's exact test, *p* < 0.05). ***D–F***, The percentage of c-Fos-positive cells was quantified for each animal individually, and the values were compared with the control group. Again, the percentage of c-Fos-positive AcD cells from the 5 d experimental group was ∼2 times larger than in the control group (Wilcoxson rank sum test, *p* < 0.05).

As described in the Materials and Methods section, we used two different analyses for assessing a potential biological relevant difference in the percentage of c-Fos-positive cells. First, we pooled all AcD or non-AcD cells from different animals of one experimental setting and calculated the odds ratio, effect size, and *p* value based on a contingency table. We defined biological relevance if the effect size was >0.2 (which corresponds to at least a small effect) or if the odds ratio was >2 (which means that the probability of an event occurring in one group is at least twice as high as the probability of it occurring in the other group). Odds ratios and effect sizes are reported in Figure 3*A*–*C*. As a second analysis, we pooled the cells from one animal to calculate the percentage of c-Fos-positive cells and compared medians between control and experimental animals (Fig. 3*D–F*). Only if the data were biologically relevant and both the contingency table and the group comparison were statistically significant, we report the data as a meaningful result.

### The increased percentage of c-Fos-positive cells in the ventral hippocampus on Day 5 of learning

First, we pooled all stained cells from the medioventral hippocampus of all animals of each experimental paradigm to assess the overall percentage of AcD and non-AcD cells expressing c-Fos (Fig. 3*A*–*C*). In general, ∼20% of cells showed c-Fos expression above the background as described in the previous paragraph (Fig. 3*A–C*). By our definition of biological relevance, the percentage of cells expressing c-Fos in animals performing the m-maze task was not different from control animals on Day 3 and Day 7 (odds ratios < 2 and effect sizes < 0.2). However, on Day 5, the percentage of c-Fos-expressing cells was significantly increased by a factor of ∼2 in AcD cells compared with control animals (odds ratio 2.38, effect size 0.18, *p* = 0.002, Fisher's exact test). In non-AcD cells, biological relevance did not meet our criteria on Days 5 and 7, despite a statistically significant higher c-Fos expression in experimental animals (odds ratio 1.62, effect size 0.11, *p* = 0.008 and odds ratio 1.63, effect size 0.11, *p* = 0.009). To further quantify the expression levels, we investigated each animal individually and compared the median values between experimental animals and control animals (Fig. 3*D*–*F*). Similar to the pooled data, the median percentage of c-Fos-expressing cells on Day 3 and Day 7 did not change by the memory task, but the percentage in AcD cells on Day 5 was ∼1.5-fold higher compared with control animals (percentage c-Fos positive, median and [confidence interval]; control, 21.4 [16.7, 27.3]; experiment, 34.9 [22.7, 50.0]; Mann–Whitney *U* test, *p* = 0.021). Interestingly, the percentage of c-Fos-positive cells was particularly low in animals that did not learn the task (Fig. 3*D*, red circles).

### The increased percentage of c-Fos-positive cells in the dorsal hippocampus during learning

**Figure 4.**
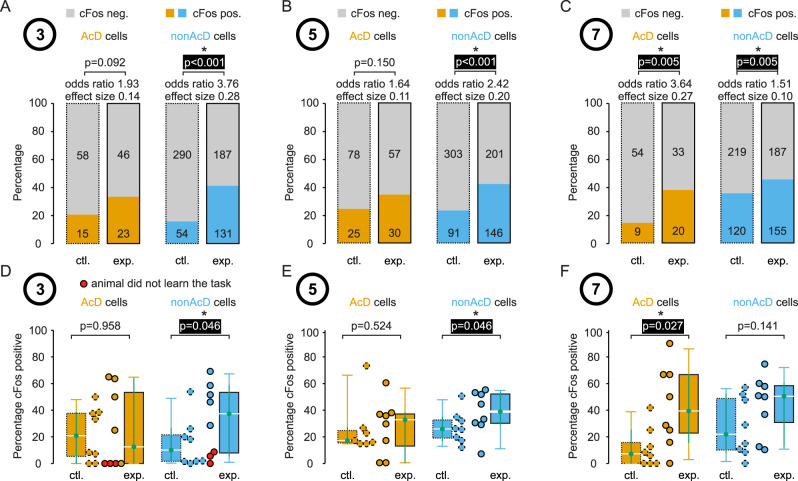
Quantification of cells expressing c-Fos in the dorsal hippocampus. Cells from three different experimental groups are analyzed: animals performed the behavior task for 3 d (***A***), for 5 d (***B***), or for 7 d (***C***). All cells from eight animals from each group where pooled and the percentage of c-Fos-positive cells quantified. Respective control groups were handled and present in the experimental room but did not perform the behavioral task. The percentage of c-Fos-positive non-AcD cells from the 3 and 5 d experimental group was ∼2.6 and ∼1.8 times larger than in the control group and the percentage of c-Fos-positive AcD cells from the 7 d experimental group was ∼2.6 larger than in the control group (Fisher's exact test, *p* < 0.05). ***D–F***, The percentage of c-Fos-positive cells was quantified for each animal individually, and the values were compared with the control group. Again, the percentage of c-Fos-positive non-AcD cells from the 3 and 5 d experimental group and the percentage of c-Fos-positive AcD cells from the 7 d experimental group was larger than in the control group (Wilcoxson rank sum test, *p* < 0.05).

Next, we pooled all cells from the dorsal hippocampus to assess the percentage of AcD and non-AcD cells expressing c-Fos during the course of the m-maze task (Fig. 4). In contrast to the medioventral hippocampus, the dorsal hippocampus of mice performing the spatial memory task showed a pronounced increase in the percentage of c-Fos-expressing cells compared with control animals (Fig. 4*A*–*C*). Throughout the learning process, we observed a shift from increased percentage in non-AcD cells at earlier stages (Days 3 and 5, odds ratio 3.76 and 2.42, effect size 0.28 and 0.20, Fishers exact test, *p* = 3.0 × 10^−13^ and 4.4 × 10^−8^) to increased percentage of c-Fos-positive cells in AcD cells on Day 7 (odds ratio 3.64, effect size 0.27, *p* = 5.0 × 10^−3^) compared with control animals (Fig. 4*A*–*C*). Again, we quantified data from individual animals and compared the different groups and time points (Fig. 4*D*–*F*). Similar to the results reported above, the median percentage of c-Fos-positive cells in non-AcD cells was increased on Day 3 and 5 (*p* = 0.046 and 0.046), while in AcD cells, it was increased on Day 7 (*p* = 0.027) when compared with control animals (median percentage of c-Fos-positive cells [confidence interval], non-AcD cells Day 3, control, 10.1 [1.5, 26.0]; experiment, 37.4 [5.6, 58.6]; non-AcD cells Day 5, control, 25.3 [16.7, 34.3]; experiment, 38.4 [28.6, 52.6]; AcD cells Day 7, control, 7.1 [0, 25.0]; experiment: 39.3 [15.8, 66.7]). Similar to the medioventral hippocampus, the percentage of c-Fos-positive cells in the dorsal hippocampus was particularly low in animals that did not learn the task (Fig. 4*D*, red circles).

### Decreased AcD occurrence during the learning process

**Figure 5.**
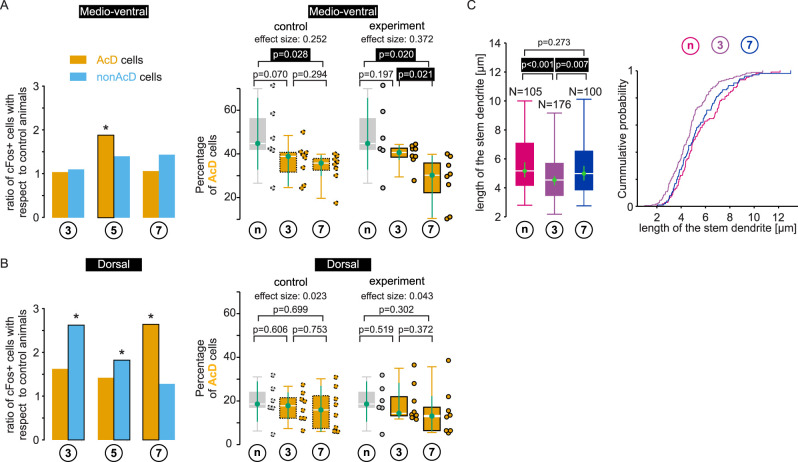
Summary of the c-Fos expression and quantification of the percentage of AcD cells. ***A***, Quantification of the medioventral hippocampus. Left panel, Ratio of c-Fos-positive cells in experimental animals with respect to control animals for the different groups. Right panel, The percentage of total number of AcD cells, irrespective of c-Fos expression in control animals (left) and experimental animals (right) from the 3 and 7 d experimental group compared with completely naive animals. AcD percentages from control animals and the experimental animal from the 7 d group are different from naive animals. Additionally, AcD percentages from the experimental 7 d group are different from the experimental 3 d group (Kruskal–Wallis test, followed by a Dunn–Bonferroni post hoc test, *p* < 0.05). ***B***, Similar to panel ***A*** but for the dorsal hippocampus. Although c-Fos expression in experimental animals is increased in non-AcD cells for the 3 and 5 d group and in AcD cells from the 7 d group (left panel), the percentage of AcD cells does not change over the course of the experiment in the dorsal hippocampus (right panel, Kruskal–Wallis test, *p* > 0.05). ***C***, The length of the stem dendrite varies over the course of the experiment. Median lengths (left panel, Kruskal–Wallis test, followed by a Dunn–Bonferroni post hoc test) and cumulative distributions (right panel) are smaller and left-shifted, respectively, for AcD cells from the 3 d experimental group.

It is presently not known whether somatic and dendritic axon locations are stable cell properties or whether learning processes could lead to changes of the axon origin location along a basal dendrite. This in turn could lead to a change in the ratio of AcD to non-AcD cells. As shown in the previous paragraphs, AcD and non-AcD cells showed different c-Fos expression profiles on different days along the learning curve (summarized in Fig. 5*A*,*B*, left panels). We therefore looked at the percentage of AcD cells during the course of the learning paradigm. Interestingly, the percentage of AcD cells in the medioventral hippocampus decreased during the observation period. Comparison of control animals to completely naive animals, i.e., animals that were not handled in any way, showed that handling and passive attendance in the experimental room already decreased the percentage of AcD cells in the medioventral hippocampus. Exact values were as follows: Kruskal–Wallis test, *p* = 0.050 for control, *p* = 0.016 for animals performing the m-maze task, followed by a Dunn–Bonferroni post hoc test, *p* = 0.028 for naive versus control animals at Day 7 and *p* = 0.020 for naive versus animals performing the m-maze task at Day 7, and *p* = 0.021 for Day 3 versus Day 7 (Fig. 5*A*; percentage control, naive, 44.8 [33.1, 65.4]; Day 3, 38.9 [25.5, 41.0]; Day 7, 35.8 [30.1, 39.1]; percentage experiment, naive, 44.8 [33.1, 65.4]; Day 3, 40.6 [37.5, 43.6]; Day 7, 30.2 [11.1, 38.9]). In the dorsal hippocampus, in contrast, the AcD percentage did not change by handling or during the learning paradigm (Kruskal–Wallis test, *p* = 0.859 for control, *p* = 0.057 for animals performing the m-maze task; Fig. 5*B*).

### Changes in AcD stem dendrite length during learning

To examine whether the decline in the percentage of AcD cells is associated with a shift of the axon along the dendrite, we measured the length of the stem dendrites (see Materials and Methods; Fig. 2*A*). Mice were analyzed on Days 3 and 7 of the learning process as these were the days on which we had observed the largest differences in AcD versus non-AcD cell numbers. Interestingly, the length of the stem dendrites in naive animals and in animals after Day 7 were similar, whereas stem dendrite length was significantly shorter after Day 3 compared with naive or Day 7 animals (Kruskal–Wallis test *p* = 2.2 × 10^−4^, followed by a Dunn–Bonferroni post hoc test, naive vs Day 3, *p* = 1.1 × 10^−4^; Day 7 vs Day 3, *p* = 0.007; Fig. 5*C*; stem dendrite length in µm: naive, 5.2 [4.8, 5.7]; Day 3, 4.5 [4.3, 4.8]; Day 7, 5.0 [4.5, 5.5]).

## Discussion

Hippocampal pyramidal cells can be classified into two different morphologies, AcD and non-AcD cells, respectively, and these structural differences have electrophysiological and functional implications (Thome et al., 2014; Hodapp et al., 2022; Stevens et al., 2024; Han et al., 2025). Taking c-Fos expression as a marker of recent plasticity, we show that prolonged learning over 7 d involves different time courses of activity in these two cell types. In particular, AcD and non-AcD cells showed different plasticity on different days along the learning curve (summarized in Fig. 5*A*,*B*, left panels). In the medioventral hippocampus, the number of AcD cells expressing c-Fos peaked after 5 d of training on the m-maze, i.e., when mice had learned parts of the task but still improved in performance. Cells with somatic axon origin (non-AcD cells) showed no increase in c-Fos expression. In the dorsal hippocampus, the time course of learning-related activity differed between both cell types. In non-AcD cells, the c-Fos expression increased on Days 3 and 5 in non-AcD cells, while AcD cells highest levels of c-Fos on Day 7, when progress in task performance had ended. Apart from c-Fos expression, the percentage of AcD cells changed over time. Compared with naive mice (no handling or exposure to the experimental room), the percentage of AcD cells in the medioventral hippocampus decreased constantly over the course of the learning experiments, indicating that the location of the axon origin is plastic.

### Different c-Fos activity of AcD and non-AcD cells in the medioventral and dorsal hippocampus

As stated above, c-Fos expression in the medioventral hippocampus was only increased in AcD cells and only on Day 5. At this stage, most of the mice had learned the rule of the inbound trial. The success rate for the outbound trial, however, had still not reached its maximum. The outbound trials require additional working memory when compared with inbound trials. The retrieval of working memory and consolidated memory traces are both supported by SPW-R complexes (Diba and Buzsáki, 2007; Jadhav et al., 2012). Interestingly, AcD cells show preferential activation during ripple oscillations (Hodapp et al., 2022), making it plausible that more AcD cells are recruited during a learning phase in which animals learn to use working memory to solve outgoing trials. However, it is unclear why c-Fos expression in AcD cells drops again on Day 7, when working memory still plays an important role. On the other hand, these changes were only observed in the medioventral hippocampus which is less involved in spatial information processing than dorsal portions of the hippocampus (Moser et al., 1993; Fanselow and Dong, 2010; Strange et al., 2014), in line with the relatively low c-Fos expression levels in these sections. The dorsal hippocampus, in contrast, is more involved in spatial information processing. Here, non-AcD cells had higher c-Fos expression levels than AcD cells, especially at early phases of the learning process when the rule of the paradigm must be learned. At the same time, c-Fos expression in AcD cells increased over the full course of the experiment, in line with the increasing load of the task for spatial working memory after learning the rule for outbound trials. Overall, these results are compatible with an increased recruitment of AcD neurons during SPW-R complexes reported previously (Hodapp et al., 2022) that are involved in memory consolidation and retrieval as well as working memory (Girardeau et al., 2009; Ego-Stengel and Wilson, 2010; Jadhav et al., 2012).

One caveat of our results might arise from two difficulties of using c-Fos as a marker for plasticity. Firstly, Fos has recently been reported to report accumulated activation of mGluRs (which is related to total excitatory input) rather than cell firing (Anisimova et al., 2023). Although excitatory synaptic input and action potential output of neurons are correlated, they are mechanistically different. Secondly, we found some immunofluorescence in virtually all pyramidal cells, despite the fact that c-Fos staining has less background noise than other immediate early gene signals (Lara Aparicio et al., 2022). Concerns about background staining were, however, largely eliminated as we found two clearly discernable peaks in our c-Fos expression levels, consistent with the observation that higher cell activity correlates with higher c-Fos expression (Nikolaev et al., 1992). While its relationship to action potential firing is complex and quantification is not trivial, c-Fos remains a widely accepted proxy for recent cellular activation and/or plasticity (Liu et al., 2012; Khan et al., 2025). Our conclusions focus on relative differences between cell types and conditions rather than absolute activity levels. Thus, we are confident that our data and analysis accurately reflect the different activity and involvement of AcD versus non-AcD cells in learning processes.

### Decrease of the percentage of AcD cells in the medioventral hippocampus

Independent from c-Fos expression, we observed a steady decrease in the percentage of AcD cells in the medioventral hippocampus throughout the learning task, while ratios between AcD and non-AcD cells remained unchanged in the dorsal hippocampus. AcD stem dendrite length decreased alongside the reduction of the AcD cell percentage, suggesting that axons move in the direction of the soma until the cells are classified as canonical non-AcD cells, including cells with stem dendrite lengths of <2 µm in our approach (see Materials and Methods; Thome et al., 2014; Hodapp et al., 2022; Stevens et al., 2024). By Day 7, median stem dendrite length increased again, suggesting that AcD cells with short-stem dendrites had turned into non-AcD cells or that the axon emanating from remaining AcD cells had moved even further away from the soma. However, without time-lapsed two–photon imaging in vivo, it is not possible to directly investigate these various possibilities. Functional and structural adaptions of the AIS during periods of learning have already been observed. The AIS can move toward the soma or away from it or change in length depending on the activity level of the neuron (Grubb and Burrone, 2010; Kuba, 2012; Jamann et al., 2021). Thus, it appears that neurons can homeostatically adapt their excitability to keep the firing rate within a physiological range (Mendez et al., 2018; Han et al., 2025). Therefore, the transition from AcD to non-AcD configuration might reflect a homeostatic mechanism during the learning paradigm. This would render the cell less excitable, especially during phases of strong perisomatic inhibition, as perisomatic inhibition is more efficient in non-AcD cells in controlling action potential generation (Thome et al., 2014; Hodapp et al., 2022).

Around Day 3, the average AcD stem dendrite length shows a significant shortening. This might reflect a transient state during the learning process where cells may be particularly active. As discussed above, AcD cells are typically more active than non-AcD cells during SPW-R than non-AcD cells (Hodapp et al., 2022), a network state that is involved in memory consolidation and memory retrieval (Girardeau et al., 2009; Ego-Stengel and Wilson, 2010; Jadhav et al., 2012). Thus, it is possible that preferred firing of AcD cells during these stages of the behavioral paradigm induces a transition of AcD cells to non-AcD cells. This could normalize overall neural activity during SPW-Rs and thus constitute a mechanism of homeostatic plasticity. In contrast, Day 7 marks an endpoint of the learning curve, where memory consolidation has higher relevance compared with memory acquisition. At this stage, the remaining AcD cells attain longer stem dendrites making them more independent from perisomatic inhibition (Thome et al., 2014; Hodapp et al., 2022). It is interesting to note that the percentage of AcD cells did not only decrease in the group of animals performing the learning paradigm but also in the equally handled control group. Apparently, the handling process and exposure to the experimental room were sufficient to induce a shift of the axon origin.

While we found distinct changes in the percentage of AcD versus non-AcD cells in the medioventral hippocampus, the dorsal hippocampus did not show such changes. It is well established that the ventral hippocampus is more involved in social and emotional stimuli, while the dorsal hippocampus is more responsible for spatial tasks (Moser et al., 1993; Fanselow and Dong, 2010; Strange et al., 2014). The absence of changes in AcD cell occurrence in the dorsal hippocampus of the control group is in line with this notion. Possibly, social cues and social memory formation had a stronger impact on the learning paradigm as originally expected and in line with the control experiment in completely naive animals. The significant changes in the medioventral hippocampus might go hand in hand with processing of social cues and interactions. Finally, we note that asymmetries between hemispheres may be a confounding factor for the observed differences in midventral versus dorsal sections. In order to make optimal use of the animals and to reduce variance, we used the right hemisphere for analysis of the dorsal hippocampus and the left hemisphere for the medioventral hippocampus. We cannot exclude, however, that there is a left–right asymmetry in plasticity of the axon origin. For example, neurons in the right hemisphere of mice tend to have more stable synapses than those in the left hemisphere (El-Gaby et al., 2015).

In summary, our work shows that AcD cells and non-AcD cells exhibit different activity (measured by c-Fos expression) during a memory task involving the formation, consolidation, and retrieval of spatial (and possibly also social) memory. It indicates that AcD and non-AcD cells undergo morphological changes and even transitions between both morphologies during a prolonged, complex learning task. These changes depend on the type and stage of the respective learning process and on the location along the dorsoventral axis of the hippocampus. In our present study, we compared c-Fos expression of AcD versus non-AcD cells in a groups-wise approach. Without tracking the axonal morphology of individual cells over several days, we cannot distinguish between two different possibilities: either AcD and non-AcD cells actually differ in their c-Fos expression patterns, or cell type-specific changes in c-Fos expression lead to morphological transitions between AcD and non-AcD properties. Future research that directly assesses the activity and morphology of individual cells in vivo over several days during a learning paradigm could help to understand the causal relations between plastic changes in neuronal morphology and function.

## Synthesis

Reviewing Editor: Deanna Smith, University of South Carolina Decisions are customarily a result of the Reviewing Editor and the peer reviewers coming together and discussing their recommendations until a consensus is reached. When revisions are invited, a fact-based synthesis statement explaining their decision and outlining what is needed to prepare a revision will be listed below. The following reviewer(s) agreed to reveal their identity: NONE. Note: If this manuscript was transferred from JNeurosci and a decision was made to accept the manuscript without peer review, a brief statement to this effect will instead be what is listed below.

Reviewers were enthusiastic about this manuscript, commenting on the clear writing, the acceptable rebuttal to previous reviews and the careful statistics. The idea that AcD and nonAcD neurons may transform into one another during learning is a novel and intriguing aspect of this work.

The reviewers had a few comments related to interpretations of the data that need to be addressed: indicated the following changes should be made:

1. There should be more details about the M-Maze paradigm. What is the consequence if the mouse goes to a wrong arm? Is there a punishment, e.g. time out? After a wrong choice, is the mouse put to the starting point to start a new trial? If not, does the current trial start from the wrong arm or the last visited correct arm, in another word, does the mouse have to finish the current trial (go back to the intersection to choose again to get the reward) before the next trial begins?

2. Figure 5D and the corresponding text in the results part do not make sense. If the nonAcD cells turn back into AcD cells, we should see increase in percentage of AcD cell from 3-day to 7-day group. The shortening and lengthening of the stem dendrite in different phases could happen in the same or different groups of neurons. The authors should not speculate it when there is no supporting evidence (e.g. 2-photon in vivo imaging tracked day by day). Thus, I suggest this figure and the corresponding text to be removed.

3. The authors propose that AcD and nonAcD neurons may interchange during the learning task. However, this conclusion is based on population-level analyses rather than live imaging. One possible confounding factor is the population of GFP-expressing cells itself. If the number of neurons expressing GFP under the Thy1 promoter changes in response to learning, this could alter the observed proportions of AcD and nonAcD cells without any actual transitions occurring. An additional analysis-such as quantifying the proportion of GFP-positive neurons per unit area-might help to address this concern.

4. At the end of the manuscript, the authors write:

"In summary, our work shows that AcD cells and nonAcD cells exhibit different activity (measured by cFos expression) during a memory task involving the formation, consolidation, and retrieval of spatial (and possibly also social) memory."

If AcD and nonAcD neurons are indeed capable of interchanging, then two possibilities must be considered: (a) that AcD and nonAcD cells truly differ in their cFos expression patterns, or (b) that changes in cFos expression (neuronal activation) drive morphological transitions between AcD and nonAcD states. A discussion of this point should be added.

5. In Hodapp (2022), AcD neurons were shown to be preferentially active during SPW-R. This raises the question: were these cells originally AcD neurons, or could they have been nonAcD neurons that transitioned into AcD in response to high activity? If relevant, please expand the Discussion to address this point.

3. Please use color-blind friendly pseudo-color images in Figure 2A, B and C, e.g. green/magenta, yellow/blue or red/cyan instead of green/red.

## Author Response

Dear Dr. Smith, dear Reviewers, Thank you for re-reviewing our manuscript 'Variation in the involvement of hippocampal pyramidal cell subtypes in spatial learning tasks'. We are pleased to see that the revised version of our manuscript was received with much more enthusiasm by the reviewers. We have carefully read their comments and have made the appropriate changes to the manuscript and figures. In the following, you will find a point-by-point response to their concerns.

1. There should be more details about the M-Maze paradigm. What is the consequence if the mouse goes to a wrong arm? Is there a punishment, e.g. time out? After a wrong choice, is the mouse put to the starting point to start a new trial? If not, does the current trial start from the wrong arm or the last visited correct arm, in another word, does the mouse have to finish the current trial (go back to the intersection to choose again to get the reward) before the next trial begins? We thank the reviewers for this important comment and apologize that we had not well described the m-maze paradigm in the previous versions of the manuscript. We have made the appropriate changes to the methods section on page 5, lines 10f and 17ff (see highlighted changes in the 'track changes' document) 2. Figure 5D and the corresponding text in the results part do not make sense. If the nonAcD cells turn back into AcD cells, we should see increase in percentage of AcD cell from 3-day to 7-day group. The shortening and lengthening of the stem dendrite in different phases could happen in the same or different groups of neurons. The authors should not speculate it when there is no supporting evidence (e.g. 2-photon in vivo imaging tracked day by day). Thus, I suggest this figure and the corresponding text to be removed.

We have removed the panel and the corresponding text as suggested (page 11, lines 4ff and 15, lines 37ff, see highlighted changes in the 'track changes' document).

3. The authors propose that AcD and nonAcD neurons may interchange during the learning task. However, this conclusion is based on population-level analyses rather than live imaging. One possible confounding factor is the population of GFP-expressing cells itself. If the number of neurons expressing GFP under the Thy1 promoter changes in response to learning, this could alter the observed proportions of AcD and nonAcD cells without any actual transitions occurring. An additional analysis-such as quantifying the proportion of GFP-positive neurons per unit area-might help to address this concern.

We are very grateful for this important comment. We have added a quantification of the number of Thy-1/GFP positive cells within the different experimental groups (Figure 2F, text on page 6, lines 31ff, page 8, lines 33ff, and 14, lines 37ff, see highlighted changes in the 'track changes' document). Cell numbers within the groups are similar, and no statistical difference could be observed, indicating that Thy-1/GFP expression is not correlated to the behavioral paradigm.

4. At the end of the manuscript, the authors write: "In summary, our work shows that AcD cells and nonAcD cells exhibit different activity (measured by cFos expression) during a memory task involving the formation, consolidation, and retrieval of spatial (and possibly also social) memory." If AcD and nonAcD neurons are indeed capable of interchanging, then two possibilities must be considered: (a) that AcD and nonAcD cells truly differ in their cFos expression patterns, or (b) that changes in cFos expression (neuronal activation) drive morphological transitions between AcD and nonAcD states. A discussion of this point should be added.

This is a very important point and we thank you for pointing that out. Accordingly, we have included this in our discussion (14, lines 2ff, see highlighted changes in the 'track changes' document).

5. In Hodapp (2022), AcD neurons were shown to be preferentially active during SPW-R. This raises the question: were these cells originally AcD neurons, or could they have been nonAcD neurons that transitioned into AcD in response to high activity? If relevant, please expand the Discussion to address this point.

Thank you very much for this valuable point of discussion. We have included it on page 13, lines 7ff (see highlighted changes in the 'track changes' document).

6. Please use color-blind friendly pseudo-color images in Figure 2A, B and C, e.g. green/magenta, yellow/blue or red/cyan instead of green/red.

We have edited the image and now use the same colorblind-friendly colors that we use in the other images.

We hope that with these changes our manuscript is now ready for publication in eNeuro.

Best regards, Martin Both and Nadja Sharkov (for all authors)
